# Atypical Phenotypes of Mitochondrial Cristae Architecture in Selected Human Aging Striated Muscles—Transmission Electron Microscope Studies

**DOI:** 10.3390/life16040658

**Published:** 2026-04-13

**Authors:** Paulina Felczak

**Affiliations:** Department of Neuropathology, Institute of Psychiatry and Neurology, 02-957 Warsaw, Poland; pfelczak@ipin.edu.pl

**Keywords:** atypical mitochondria, damaged cristae, striated muscle, aging, ultrastructure, TEM

## Abstract

The diversity of mitochondria ultrastructure in human aging striated muscles is presented in relation to the complexity and variability of the cristae architecture and in relation to the environment of mitochondrial occurrence in the muscle fiber on the example of the orbicularis oculi. Ultrastructure analysis of mitochondria in muscles was performed using a transmission electron microscope. The studies revealed the presence of mitochondria of various sizes and unexpected shapes, and also modifications of the cristae architecture which included the occurrence of different configurations of membranes. In some mitochondria, instead of cristae, crystalline inclusion bodies and granules resembling calcium deposits were found. The range of diversity of the studied morphotypes of mitochondria exceeds the algorithms for the morphology of these organelles presented in the literature to date. This diversity of mitochondria should probably be viewed as a manifestation of evolution from the classical cristae architecture to a wide range of forms of mitochondria corresponding to the current environmental conditions of the muscle fiber.

## 1. Introduction

Mitochondria are essential eukaryotic organelles that originate from endosymbiotic alphaproteobacteria [1]. These organelles consist of four compartments: (I) the matrix containing the mitochondrial genome (mtDNA), its transcription and translation mechanisms, and enzymes involved in β-oxidation of fatty acids and the citric acid cycle; (II) the inner membrane (IM) and its respiratory chain complexes (oxidative phosphorylation, OXPHOS), which generate adenosine triphosphate (ATP) for cellular processes; (III) the outer membrane (OM) containing proteins involved in cell death and protein import and voltage-dependent anion channels (VDACs); and (IV) the intermembrane space (IMS), which contains OXPHOS intermediates [2]. The OM is smooth and faces the cytosol. The IM is topologically heterogeneous and includes two distinct regions: the inner limiting membrane (IBM) adjacent to the OM and the inner crista membrane (ICM)—the region of the IM that forms invaginations towards the matrix called cristae. The tight junctions between the IBM and cristae membranes are called crista junctions (CJs). The formation and maintenance of these junctions is crucial for the organization and function of mitochondria. Furthermore, it is known that a large, heterooligomeric protein complex is located at cristae junctions. This complex is called MICOS (mitochondrial contact site and cristae organizing system) and is crucial for maintaining the characteristic architecture of mitochondria [3,4,5,6]. Several evolutionarily conserved subunits of the MICOS complex have been identified, from Mic10 to Mic60. These two subcomplexes are anchored in the inner membrane and expose their domains to the intermembrane space [3,7]. Reduced expression levels of MICOS subunits cause drastic changes in IM morphology and lead to a significantly reduced number of CJs. As a result, the cristae membranes separate from the rest of the IM and appear as stacks in the matrix [8]. The balance between the two opposing processes, fission and fusion, regulates the number, size and position of mitochondria in the cytoplasm [6,9]. As important structures involved in cellular metabolism, mitochondria often undergo adaptive changes in morphology, components and functions in response to various environmental stresses and cellular demands. This phenomenon of trait variability is termed a heterogeneity of mitochondria [10]. Dysregulation of mitochondrial dynamics is one of the key mechanisms of various disorders [11]. Changes in physiological energy states and oxygen availability especially affect the morphology of the cristae and cristae junctions, suggesting that the formation of these cristae junctions is a dynamic process that promotes cristae remodeling as an adaptive mechanism dependent on the needs and metabolic state of the cell [4]. The aim of the study was to present the diversity of mitochondrial morphology found in aging striated muscles (exemplified by the orbicularis oculi) in relation to the complexity and variability of the cristae architecture and in relation to the specific environment of mitochondrial occurrence.

## 2. Materials and Methods

Electronograms from the archives of the Electron Microscopy Laboratory at the Department of Neuropathology, Institute of Psychiatry and Neurology in Warsaw were used for this study. These were ultrastructure images of aging striated muscle, represented by the orbicularis oculi muscle. Randomly selected areas of striated muscle visible in the electronograms belonged to patients of various ages (the age range of the selected muscles included the following groups: 50+ (51, 57), 60+ (60, 62, 63), and 70+ (74 and 79)), and were the result of routine procedures for preserving material for ultrastructural studies.

These procedures included pre-fixing tissue fragments in a 2.5% glutaraldehyde solution in a cacodylate buffer at pH 7.4, followed by fixation in a 1% osmium tetroxide solution in the same buffer. After dehydration in a graded series of ethanol alcohols and propylene oxide, the material was embedded in Spurr resin. The chemicals used were from Merck KGaA, Darmstadt, Germany. Semithin sections were stained with toluidine blue to select the appropriate areas. Ultrathin sections were contrasted with uranyl acetate and lead citrate (Sigma-Aldrich, St. Louis and Burlington, MA, USA). Imaging was done using a JEM 1400 transmission electron microscope (TEM) (JEOL Co., Tokyo, Japan, 2008). TEM studies were performed in the Laboratory of Electron Microscopy of the Nencki Institute, supported by the project financed by the Minister of Education and Science based on contract No 2022/WK/05 (Polish Euro-BioImaging Node “Advanced Light Microscopy Node Poland”).

## 3. Results

**Figure 1:**
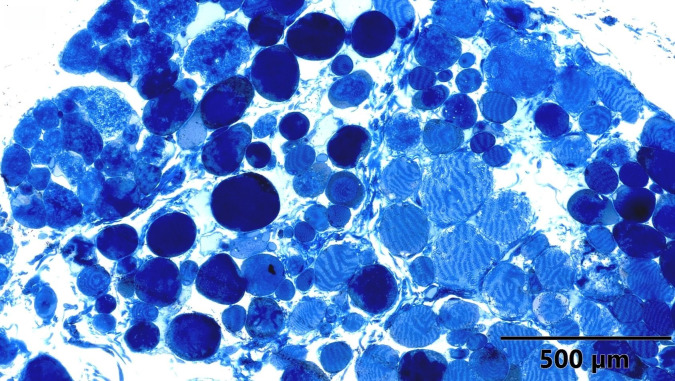
Striated muscles of different diameter and fibril density, visible in toluidine blue staining, magnification ×40.

**Figure 2:**
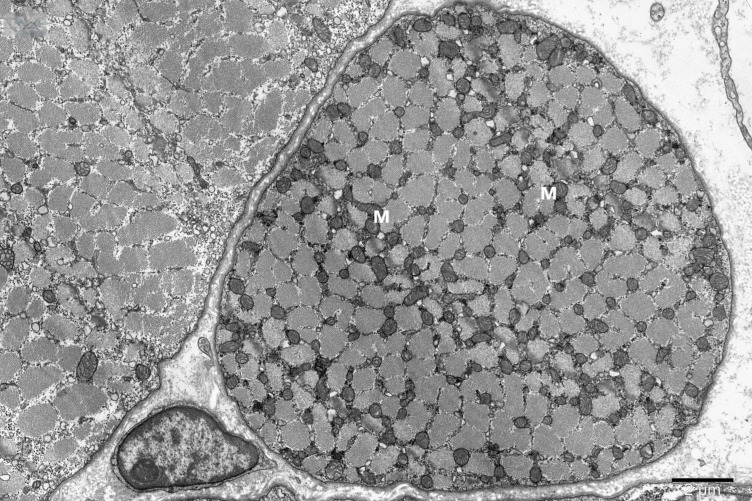
Regularly arranged intramyofibrillar mitochondria (M) in the ultrastructure of the normal striated muscle fiber, magnification ×8000.

**Figure 3:**
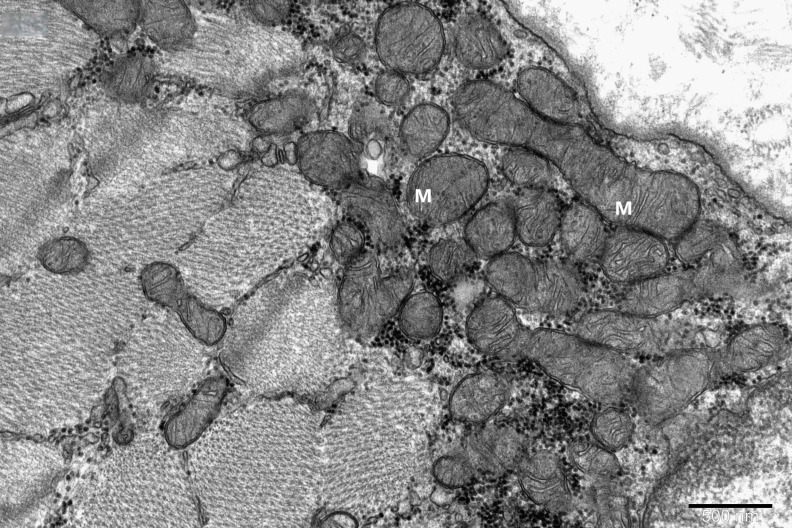
A cluster of typical subsarcolemmal mitochondria (M) in a fragment of a striated muscle fiber; magnification ×40,000.

**Figure 4:**
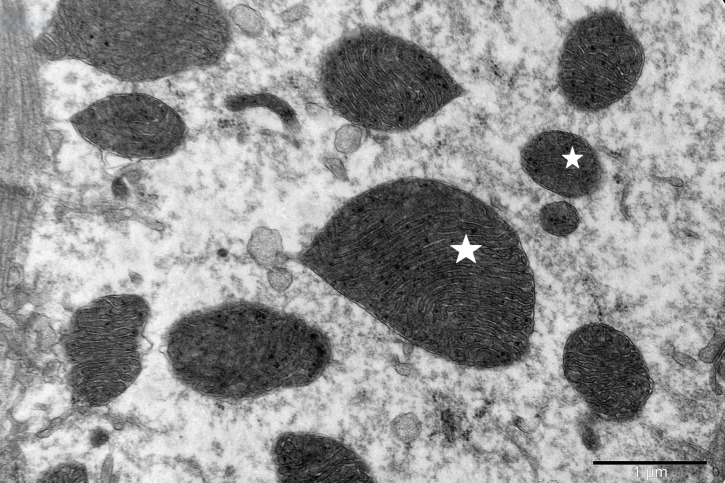
Electron-dark mitochondria (asterisk), large and ovoid or small and rounded, with numerous cristae, visible in the sarcoplasm of the muscle fiber; magnification ×30,000.

**Figure 5:**
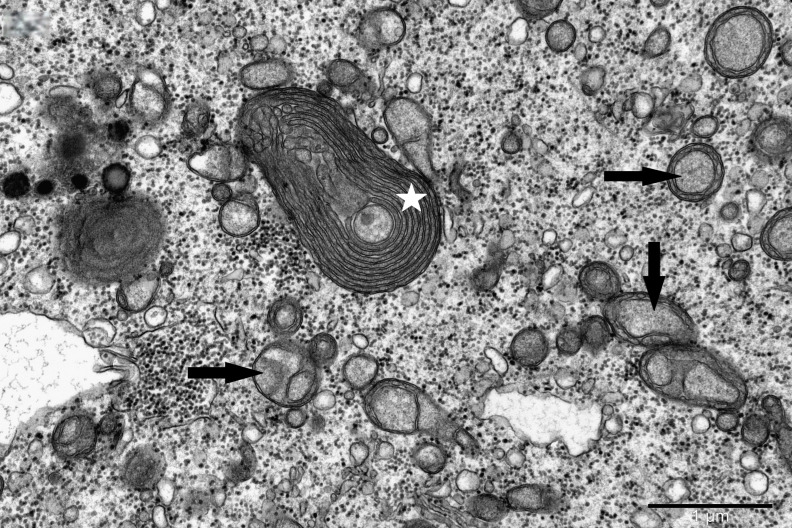
Club-shaped mitochondria with concentric inner membranes (asterisk); numerous small vesicle-like mitochondria with a visible matrix (arrow) in a muscle fiber; magnification ×30,000.

**Figure 6:**
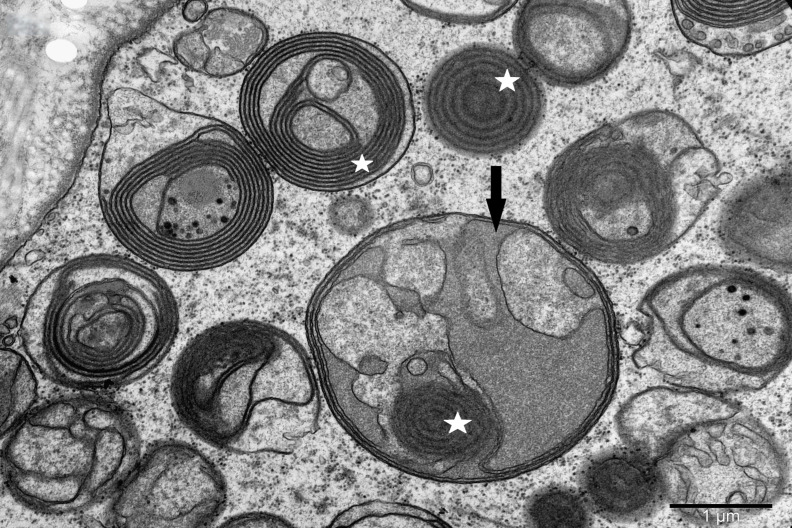
Group of large, vesicle-like mitochondria filled with membrane coils of various configurations (asterisk). Giant mitochondria (arrow) with a heterogeneous matrix; magnification ×25,000.

**Figure 7:**
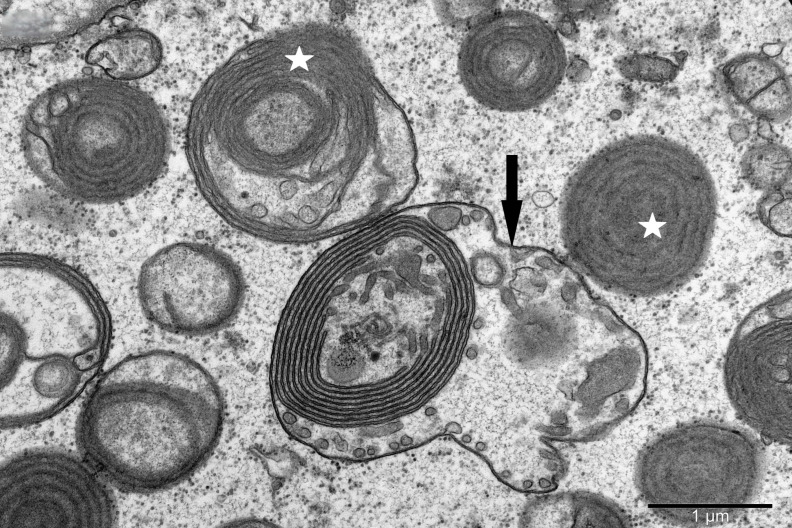
Vesicle-like mitochondria (asterisk) with concentric membrane arrays and giant mitochondria with a membrane fold resembling “amoeba pseudopodia” (arrow); magnification ×30,000.

**Figure 8:**
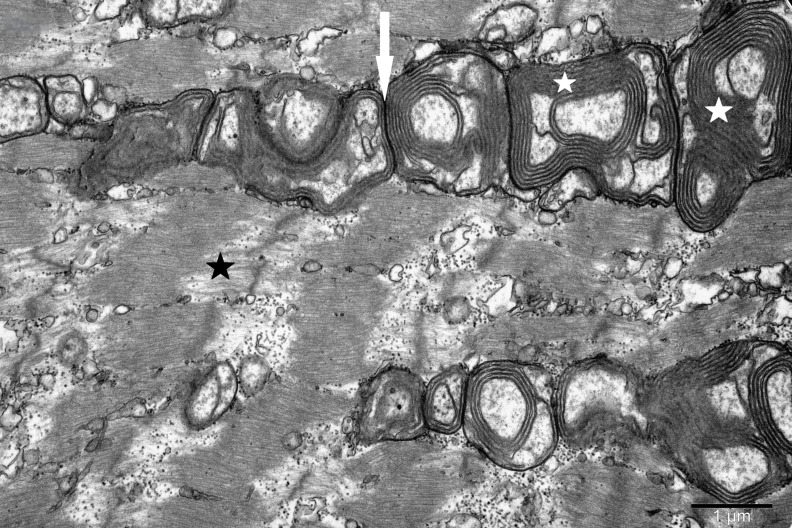
Tightly packed atypical mitochondria (arrow) with concentric membrane invaginations (white asterisk) visible in a fiber with sparse myofilaments (black asterisk); magnification ×20,000.

**Figure 9:**
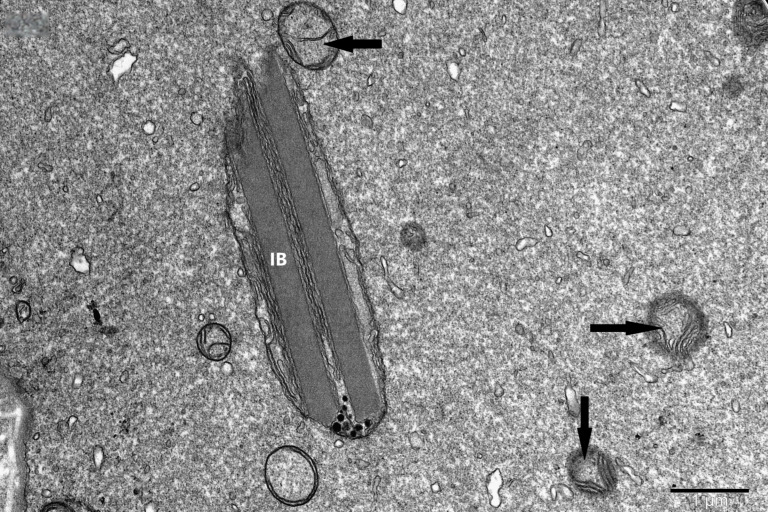
Rod-shaped mitochondria filled with crystalline inclusion bodies (IB) and containing a cluster of dark granules. Small mitochondria with simplified structure (arrow); magnification ×20,000.

**Figure 10:**
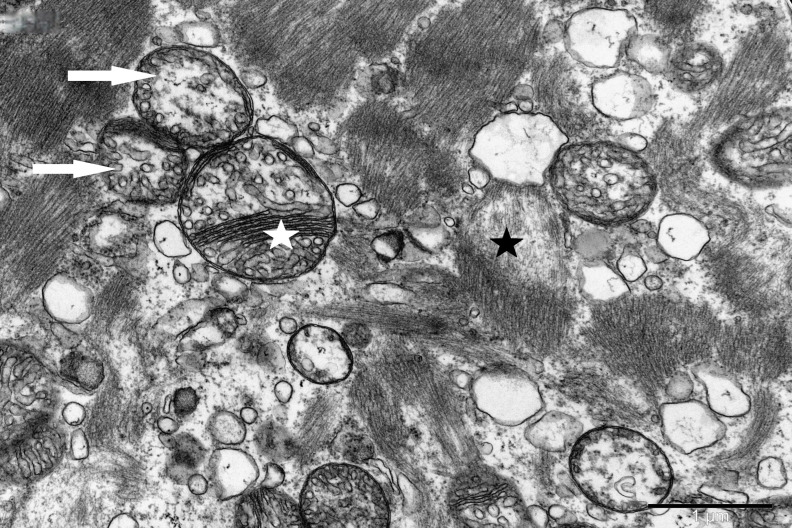
Round mitochondria with a tubular–lamellar (white asterisk) or sparsely tubular (arrow) organization of the inner membranes, surrounded by scattered myofilaments (black asterisk); magnification ×30,000.

**Figure 11:**
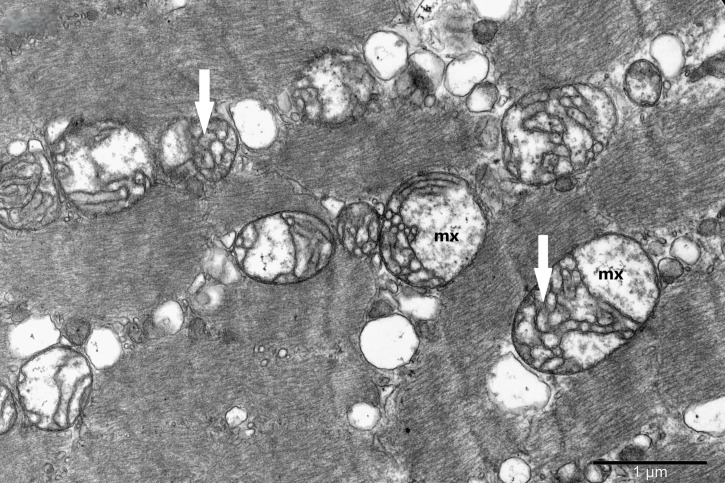
Mitochondria with openwork cristae architecture (arrow) and bright matrix (mx) in a striated muscle fiber; magnification ×30,000.

To study the ultrastructure of striated muscle mitochondria, randomly selected fragments of aging striated muscles (orbicularis oculi) from three age groups (50+, 60+ and 70+) were used. The analyzed striated muscles occurred in groups and were visible on semithin preparations in toluidine blue staining. These muscles differed in diameter and fibril density, which in the microscopic image corresponded to lighter and darker sectors of muscles (Figure 1). In ultrastructural studies, mitochondria that were observed in fibers differed in appearance. Two groups of mitochondria were characterized by a fairly typical structure. In the first group, mitochondria occurred in regular arrangements, as intramyofibrillar organelles, in normal fibers (Figure 2). They were round or elongated in cross-section, with visible cristae systems and surrounded by double membranes (Figure 3). The second group consisted of mitochondria with high electron density and numerous cristae filling their interiors. These mitochondria were mostly large and ovoid or small and rounded (Figure 4). The next morphotypes of mitochondria studied differed significantly in appearance from these basic forms. They were found mainly in sectors of fibers characterized by disturbed ultrastructure, e.g., by disorganization of myofibrils or loss of myofilaments (Figure 5, Figure 6, Figure 7, Figure 8, Figure 9, Figure 10 and Figure 11). Micrographs of normal-looking mitochondria (Figure 3 and Figure 4) in the examined fibers were compared with ultrastructural images of mitochondria with atypical morphology. Deformed mitochondria presented different phenotypes. For example, there were club-shaped mitochondria with a concentric, dense system of internal membranes (Figure 5). They occurred in the sarcoplasm, surrounded by miniature round mitochondria with a bright matrix. Special attention was paid to mitochondria with an extreme appearance. These structures represented phenotypes in the form of giant vesicles or small vesicles with a heterogeneous matrix (Figure 6). Inside these giant mitochondria, membranes with circular or semicircular arrangements were visible. From time to time, dark granules resembling calcium deposits were also present. Deformations of the outer membranes of giant vesicular mitochondria sometimes looked like “amoeba pseudopodia” (Figure 7). Other mitochondrial phenotypes resembled partially concave vesicles with concentric inner membranes and bright areas corresponding to the matrix. They formed compact rows surrounded by scattered myofilaments (Figure 8). An interesting finding was rod-shaped mitochondria filled with crystalline inclusions and containing dark granules resembling calcium deposits. These mitochondria were adjacent to small mitochondria with simplified morphology, which were observed in the regions of fibers devoid of myofibrils (Figure 9). Examples of other morphotypes were rounded mitochondria with a tubular–lamellar or sparsely tubular organization of the internal membranes (Figure 10) occurring in the surroundings of disturbed myofibrils. A distinct phenotype was presented by mitochondria, in which the cristae formed structures resembling an openwork mesh with membrane defects, and with a bright matrix visible there (Figure 11). The phenotypes of deformed mitochondria in the age groups we studied were comparable. They did not differ significantly.

### Microscopic Photos

In the presented photographic documentation, the observed changes in the ultrastructure of mitochondria in aging striated muscle are illustrated using the orbicularis oculi muscle (Figure 1, Figure 2, Figure 3, Figure 4, Figure 5, Figure 6, Figure 7, Figure 8, Figure 9, Figure 10 and Figure 11).

## 4. Discussion

Mitochondria are highly plastic and dynamic organelles, crucial for cellular metabolism [11]. Among the four compartments of mitochondria, i.e., the outer membrane (OM), inner membrane (IM), matrix and intermembrane space (IMS), the invaginations of the inner membrane, i.e., mitochondrial cristae, deserve special attention [1,12,13]. Cristae are the main sites of electron transfer [14] and are responsible for cellular ATP production via oxidative phosphorylation (OXPHOS) [15]. The maintenance of the cristae structure is due to the large protein complex MICOS (mitochondrial contact site and cristae organizing system) [5] and other proteins such as the dynamin-like GTPase OPA1 (optic atrophy type 1) and the F1 F 0-ATP synthase [16,17]. Abnormal cristae architecture is associated with many disorders, indicating that their structure and dynamics are fundamental for proper mitochondrial and cellular physiology [7,11].

The research involved analysis of the ultrastructure of mitochondria in aging striated muscle fibers (exemplified by the orbicularis oculi) from several randomly selected muscles of different ages. The results of these studies revealed a surprising phenotypic diversity of mitochondria and their local accumulations. Moreover, the results obtained here do not indicate an association between the diversity of mitochondrial morphology and the age of the muscles.

The studied mitochondria were conventionally treated as populations of organelles living in the environment of aging striated muscle fibers (orbicularis oculi).

According to the Hardy–Weinberg rule, in the so-called ideal population the frequency of all alleles is constant and unchanging over time, i.e., no evolution occurs. Such a population is not subject to any influences and is in a state of equilibrium [18,19,20]. However, in nature, achieving such a state seems impossible. Assuming that populations occurring in natural conditions evolve [21,22], the populations of mitochondria in aging muscle fibers which were studied will also evolve. The manifestation of this development at the intracellular level may be the observed diversity and complexity of the morphological forms of mitochondria, or on the contrary, their simplification. In this view, this diversity of mitochondria seems to be a response to the changing conditions of the muscle fiber environment in which mitochondria occur.

Evidence for the evolution of mitochondria, since the emergence of an integrated organelle, is the continued evolutionary divergence in mitochondrial form and function. Studies of mitochondria from various eukaryotes, including unicellular and multicellular ones, have disproved the notion that mitochondria are a “single type” of organelle. Mitochondrial genomes and proteomes vary considerably across eukaryotic diversity [23]. Furthermore, both the size and number of mitochondria per cell can vary across organisms, tissues, and cells. For example, mature erythrocytes do not contain mitochondria, but a single liver cell can contain as many as two thousand [24]. In the examined mitochondrial populations in aging striated muscle (orbicularis oculi), mainly abnormal mitochondria were found. However, there were also mitochondria that resembled the norm. In the population of normal mitochondria, two morphological types were conventionally distinguished: type I with an electron-bright matrix and lamellar cristae and type II with an electron-dark matrix and dense cristae. Mitochondria of I type were round or elongated and rod-shaped. They resembled mitochondria in the orthodox state, which occur in the presence of low concentrations of ADP (adenosine diphosphate). Mitochondria of II type were round, ovoid or torpedo-shaped. They looked similar to condensed mitochondria, which appear in the presence of high concentrations of ADP [15]. The transition from the orthodox configuration to the condensed configuration is a manifestation of mitochondrial dynamics and a response to changes in their metabolic state [24]. Cristae undergo continuous cycles of membrane remodeling, and their integrity is crucial for proper mitochondrial function. Under physiological conditions, mitochondrial dynamics and changes in their morphology are observed [15]. The research carried out also shows that under conditions associated with the aging process of the fibers, a surprising change in the appearance of mitochondria can occur. During these studies, atypical mitochondria found in fibers occurred there with some repeatability as phenotypically distinct populations of organelles. They differed in size, shape, the arrangement of internal membranes and the presence/absence of inclusion bodies. Both giant and very small mitochondria were visible. There were mitochondria with club-shaped, vesicular, amoeboid shapes, as well as multiform or partially flattened forms. Regardless of the shape, concentrically organized internal membranes were often found in these mitochondria.

It is known that the above-mentioned MICOS complexes, OPA1 protein and ATP synthase dimers may be regulators of the crista structure [15]. A link between these cristae proteins and mtDNA organization in mitochondria has also been demonstrated [25]. Disorders of mitochondrial membrane remodeling factors contribute to abnormal cristae architecture [16]. Loss of some MICOS components, e.g., MIC60, MIC10 and MIC19, may be associated with a complete loss of cristae connections and their disorganization. This leads to enlarged mitochondria with abnormal circular cristae, referred to as “concentric rings” or “onion-like structures”. This remodeling of membrane architecture is associated with nucleoid clustering, which is accompanied by a reduction in mtDNA transcription [25,26]. In addition, dysfunction of the F1F0 ATP synthase dimer stabilizing subunits can also cause concentric rings/structures resembling onions or balloon-shaped cristae [16,26]. In turn, a decrease in the level of OPA1 protein, its deletion or improper processing cause widening of the cristae and CJs, a decrease in the number of cristae and, in the case of impaired IM fusion, membrane fragmentation [26]. The number of mtDNA copies in mitochondria is also reduced [25].

In the examined mitochondria of the striated muscle (orbicularis oculi), the presence of deformed outer membranes and a heterogeneous matrix was also found, which may suggest the participation of other proteins in the development of these changes.

These proteins are responsible for mitochondrial dynamics: DRP1 (dynamin-related protein 1), mitofusins (MFNs), Mfn1 and Mfn2 [25], as well as chaperones and matrix proteases (mitoproteases), which, among other things, control the process of protein folding and remove damaged proteins [16,27]. Normally, cytosolic DRP1 moves to the outer membrane, where it is responsible for the correct separation of mitochondria. Its deficiency leads to the formation of the so-called mito-bulb. A bulge appears in the mito-bulb with dense cristae and an accumulation of mtDNA molecules [16,25].

During the research, rod-shaped mitochondria with inclusion bodies, probably located in the intermembrane space, were observed. Granules resembling calcium deposits were visible next to these inclusions. Similar-looking granules were present in some mitochondria with concentrically organized membranes. It is known that creatine kinase (CK) catalyzes the reversible transfer of the N-phosphoryl group from phosphocreatine (PCr) to ADP to regenerate ATP. This enzyme plays a key role in energy homeostasis in cells with periodically high, variable energy requirements, such as skeletal muscle fibers. One of the isoforms, i.e., mitochondrial creatine kinase (Mi-CK), stabilizes mitochondrial membranes. In pathological conditions, compensatory overexpression of this isoform, caused by energy deficiency, may lead to the formation of intramitochondrial Mi-CK crystalline inclusions [28]. Even short interruptions in ATP production in mitochondria are destructive to energy homeostasis. One of the causes of disturbances in this balance is calcium overload of mitochondria. Calcium then accumulates in the form of calcium phosphate granules and has a detrimental effect on the remodeling of the inner membrane. Low levels of free calcium in the matrix (<5 μM) activate dehydrogenases and enhance OXPHOS. In turn, its excess limits or inhibits ATP synthesis, which can lead to mitochondrial rupture and fragmentation [29].

In these studies, the occurrence of high structural diversity of mitochondria in aging striated muscle fibers may suggest the operation of mechanisms that control the modeling of the cristae and protect mitochondria from the processes of their disintegration. From the author’s own previous studies [30,31] and from the literature, it is known that a certain repeatability of specific morphological types of mitochondria is observed. Therefore, attempts were made to classify these organelles according to the presented phenotypes. Taking into account changes in the dynamics of the cristae, in addition to the typical “bean-shaped mitochondria”, compact, branched, nanotunnel, elongated, donut-shaped mitochondria, and megamitochondria were distinguished [13].

According to the conducted research, the observed mitochondrial morphotypes are not limited to the forms listed above, but extend this list to include vesicular mitochondria with concentric cristae or rod-shaped mitochondria with regularly shaped inclusions. Therefore, attempts to create algorithms for morphological classification of mitochondria may have practical justification. The task is difficult because it requires taking into account both intramitochondrial factors and factors of the intracellular environment, which are likely to affect the final form of mitochondria. For example, donut-shaped mitochondria may be an early marker of cellular stress. It has been shown that the production of reactive oxygen species (ROS) induced by complex I and III inhibitors resulted in a reversible transition of mitochondria from an elongated form to a donut-shaped form, which was associated with an increased level of ROS in mitochondria [13]. In turn, defects in mitochondrial fission and fusion likely contribute to the formation of megamitochondria. During stress, fusion increases mitochondrial complementation and maximizes oxidative capacity as a compensatory mechanism [11].

The analysis of mitochondrial diversity in aging striated muscle also requires consideration of the specificity of these muscles in relation to mitochondria. Active muscles produce energy in the form of work and heat, which come from reactions [32] involving ATP generated in mitochondria. The kinetic properties of fibers are controlled by myosin, which is also the basis for the classification of fiber types. Muscle contraction speed and strength are functions of myosin ATPase activity. In mammalian striated muscles, several myosin heavy chain genes are expressed. Differences in fiber structure, myosin isoform composition, gene expression pattern, and work speed of individual fibers [33] suggest that fiber mitochondria must meet special requirements. On the other hand, the unexpected phenotypes of mitochondria that are observed in these fibers may represent a way for these organelles to adapt to the environmental conditions of aging muscle fibers.

## 5. Conclusions

(a) The demonstrated diversity of mitochondrial phenotypes in selected aging striated muscles extends the scope of mitochondrial morphology algorithms presented in the literature so far to include new and unexpected morphotypes.
(b) The observed mitochondrial heterogeneity seems to be a manifestation of evolution from classical cristae architecture to forms adapted to the current conditions of the muscle fiber environment.

## Data Availability

All data important for this study are included in the article.
